# Ecological momentary assessment of sleep, pain, and opioid use among adolescents following surgery

**DOI:** 10.1093/sleepadvances/zpae039

**Published:** 2024-06-17

**Authors:** Andrew H Rogers, Jennifer A Rabbitts, Michael G Saper, Gregory A Schmale, Tonya M Palermo, Cornelius B Groenewald

**Affiliations:** Center for Child Health, Behavior & Development, Seattle Children’s Research Institute, Seattle, WA, USA; Department of Anesthesiology and Pain Medicine, University of Washington School of Medicine, Seattle, WA, USA; Department of Anesthesiology, Perioperative, and Pain Medicine, Stanford University School of Medicine, Stanford, CA, USA; Department of Orthopedic Surgery and Sports Medicine, University of Washington School of Medicine, Seattle, WA, USA; Department of Orthopedic Surgery and Sports Medicine, Seattle Children’s Hospital, Seattle, WA, USA; Department of Orthopedic Surgery and Sports Medicine, University of Washington School of Medicine, Seattle, WA, USA; Department of Orthopedic Surgery and Sports Medicine, Seattle Children’s Hospital, Seattle, WA, USA; Center for Child Health, Behavior & Development, Seattle Children’s Research Institute, Seattle, WA, USA; Department of Anesthesiology and Pain Medicine, University of Washington School of Medicine, Seattle, WA, USA; Department of Anesthesiology, Perioperative, and Pain Medicine, Stanford University School of Medicine, Stanford, CA, USA

**Keywords:** pain, depression, pediatrics—sleep and arousal

## Abstract

**Background:**

Opioids are effective for acute pain management following surgery among adolescents, yet are associated with significant negative consequences, including respiratory depression and opioid misuse. Sleep deficiency is common following surgery and extant research indicates strong cross-sectional associations between sleep deficiency and increased problematic opioid use.

**Objective:**

This study examined longitudinal associations between postsurgical sleep deficiency and opioid use among adolescents undergoing outpatient surgery. We also examined daily pain and mood as mechanisms linking previous night’s sleep deficiency and next day prescription opioid use.

**Methods:**

This prospective, observational study enrolled 106 adolescents (11–19 years) who underwent orthopedic outpatient surgery and collected pre-surgery and longitudinal measurements. Participants were 52% female, African-American (7%), American Indian/Alaska Native (7%), Hispanic (9%), Native Hawaiian or Other Pacific Islander (4%), or white, non-Hispanic (66%). Using ecological momentary assessment methods, participants reported sleep, pain, and mood in real time over the first 14 days following surgery. Postsurgical opioid use was measured using an electronic medication cap monitoring device, eCAP^TM^. Associations between variables were measured using multilevel structural equation modeling.

**Results:**

Using multi-level mediation models, pain, but not mood-mediated associations between postsurgical sleep deficiency (sleep quality, total sleep time, sleep onset latency, and wake after sleep onset) and opioid use, at both the within-person and between-person levels. Results highlight that greater previous night’s sleep deficiency (both generally and greater than a person’s mean level) was associated with higher next day pain (both generally and greater than a person’s mean level), which, in turn, was associated with higher opioid use. Furthermore, between-person total effect models provide support for sleep deficiency predicting higher opioid use.

**Conclusions:**

Our findings should be considered preliminary yet underscore the need for a comprehensive and personalized approach to postsurgical pain management and opioid use, potentially implementing interventions targeting sleep quality and quantity to reduce pain and opioid use.

**Statement of Significance**

Associations between sleep deficiency and opioid use remain poorly described in clinical populations. We evaluate pain intensity and mood symptoms as mediators underlying associations between sleep and opioid use among adolescents undergoing outpatient surgery.

On average, >300 000 opioid prescriptions are provided to the approximately 1.8 million adolescents (10–19 years) undergoing outpatient surgery in the United States each year. Prescribed opioids provide effective post-surgical pain control and are considered an important treatment option when combined with a multimodal pain regimen for acute postsurgical pain. Indeed, poorly controlled acute postsurgical pain is associated with a host of sequelae, including elevated physiologic stress response as well as delayed functional recovery, and an increased risk of developing chronic pain [1]. However, opioid use is associated with significant side effects which may delay recovery, including nausea, constipation, drowsiness, and respiratory depression, and up to 15% of children develop problematic opioid use behaviors following postoperative opioid prescription [2]. Among previously opioid-naïve patients, greater prescription opioid use is associated with greater opioid-related side effects and opioid-related risk behaviors [3]. Identifying modifiable factors associated with increased opioid use during the immediate postsurgical period may lead to interventions aimed at reducing opioid-related risk among adolescents.

Sleep deficiency is a broad construct that includes sleep deprivation (not getting enough sleep), noncircadian sleep (sleeping at the wrong time of the day), impaired sleep architecture (not achieving all required types of sleep), sleep disorders (e.g. sleep-disordered breathing, insomnia), and poor sleep quality. Among adults, sleep deficiency is common following surgery and is associated with increased pain intensity and opioid use [4]. Indeed, adult behavioral (e.g. presurgical sleep extension [5]) and pharmacological (e.g. dexmedetomidine [6], zolpidem [7]) treatments have been demonstrated in randomized clinical trials to reduce perioperative sleep deficiency and improve associated pain and reduce opioid use after surgery. Among adolescents, it is estimated that 20%–47% report significant sleep deficiency following surgery [8, 9]. While extant research has found strong associations between sleep deficiency and opioid misuse among adolescents in cross-sectional analysis of large public datasets [10, 11], limited work has examined longitudinal associations between sleep deficiency and opioid use behaviors following surgery. Raymond et al. and Mun et al. examined longitudinal associations between sleep and opioid use in participants with burn injury and sickle cell disease respectively, finding that sleep deficiency prospectively predicts opioid use [12, 13]. However, these previous studies did not use ecological momentary assessment (EMA). Adolescent sleep deficiency is modifiable using existing behavioral and pharmacological interventions. Therefore, a better understanding of how sleep deficiency impacts postsurgical opioid use may reveal new avenues for reducing postsurgical opioid use through sleep interventions.

Moreover, it is important to identify modifiable mechanisms that may maintain the sleep deficiency-opioid use relationship among adolescents. Pain experience and negative mood are two modifiable risk factors that have been separately associated with both sleep deficiency and opioid use, and may serve as mechanisms linking sleep deficiency to postsurgical opioid use [14, 15]. Indeed, research on the bidirectional associations between sleep and pain supports the direction of sleep deficiency causing pain as stronger than the direction of pain causing subsequent sleep deficiency, such that treating sleep deficiency using psychological treatments improves overall pain [14, 16]. Similarly, sleep deficiency was found to be associated with increased negative mood symptoms, including anxiety and depression [17]. Not surprisingly, increased pain experience has been linked to greater opioid use among both adolescents and adults, and the same pattern has been found for negative mood and opioid use [18–21]. Despite research demonstrating connections among sleep deficiency, pain, negative mood, and opioid use, research to date has not yet examined sleep, pain, and negative mood in real-time as modifiable, daily-level mechanisms which may contribute to greater overall opioid use among postsurgical adolescents.

To address these gaps in knowledge, we examined pain and mood as mechanisms linking the previous night’s sleep to next day’s prescription opioid use using daily diary data for 14 days following surgery. Participants were previously opioid-naïve adolescents. We hypothesized that sleep deficiency would be associated with higher prescription opioid use, and that higher pain intensity and higher depressive mood symptoms would mediate this relationship both within person as well as between participants.

## Methods

### Study design

The current study was a prospective, observational cohort study conducted at an academic pediatric hospital located in the Pacific Northwest region of the United States. This study followed the Strengthening the Report of Observational Studies in Epidemiology (STROBE) reporting guidelines for cohort studies. The current study was conducted as a part of a larger study investigating perioperative risk and protective factors associated with postsurgical opioid use and misuse and is the first publication from this dataset. This study was approved by the institutional review board at Seattle Children’s Research Institute.

### Participants

Participants included 106 adolescents meeting the following inclusion criteria: (1) 10–19 years of age, (2) internet access via smartphone, and (3) underwent outpatient sports-injury orthopedic surgery at Seattle Children’s Hospital, including one of the following surgeries: anterior cruciate ligament reconstruction, medial collateral ligament reconstruction, lateral collateral ligament reconstruction, meniscus repair, medial patellofemoral ligament reconstruction, osteochondritis dessicans debridement, shoulder, and elbow surgery. All surgeries were performed by one of the two pediatric fellowship-trained orthopedic surgeons (M.G.S. and G.A.S.) under combined general and regional anesthesia. Exclusion criteria included (1) American Society of Anesthesiologists physical status 3 or greater (severe systemic disease), (2) prior diagnosis of opioid misuse or opioid use disorder, (3) significant underlying physical or mental conditions that may prevent/interfere with completion of the study based on the assessment of the primary investigator (e.g. neurodevelopment delays), and (4) participants unable to complete questionnaires in English. One participating parent was also enrolled.

### Study procedures

Eligible participants were identified from the surgical schedule and were sent an invitation to participate in the study. Research coordinators subsequently contacted eligible participants by phone to screen interested participants and explain study procedures. Informed consent was obtained from youth (18 years and older) and parents, while youth <18 years provided assent to study procedures. Between January 2020 and January 2023 189 eligible youth were approached for potential participation, of whom 106 enrolled (56% participation). During the first 14 days immediately following surgery, self-reported survey data assessing sleep, pain, and mood, were collected four times/day (at 4-hour time intervals) while awake using Research Electronic Data Capture (REDCap, Vanderbilt University). Survey links were texted or sent to participants’ email addresses with automated reminders sent if questionnaires were not completed. Postsurgical opioid use was objectively measured using an electronic medication adherence device, eCAP^TM^. Data on surgery type and opioids prescribed (number of doses) were abstracted from the patient’s medical records. All participants were prescribed oxycodone, no other opioids were prescribed. The retention rate through follow-up was 95%, with 5 participants dropping out. Overall, youth completed 86% (5474 entries out of 6381) of EMA surveys sent to them during the 14-day monitoring period.

## Measures

### Sociodemographics

Parents reported on their child’s biological sex (male/female), age, race, and ethnicity, as well as household income level. Race and ethnicity options included (1) American Indian/Alaska Native, (2) Native Hawaiian or Other Pacific Islander, (3) Hispanic, (4) African-American, (5) White, and (6) Other.

### EMA of sleep, pain, and mood

Beginning on the day after surgery, adolescents completed electronic EMA surveys daily during each of four predesignated time intervals (7–11 am, 11 am–3 pm, 3–7 pm, and 7–9 pm) via REDCap for a 14-day period on their smartphones. EMA included ratings of current pain intensity and mood (11-point numeric rating scales [NRS, 0–10] with higher ratings indicating higher pain and better mood, respectively). Sleep parameters were collected each morning via REDCap sleep diary. Adolescents were asked to record previous evening’s sleep including time getting into bed, time when they fell asleep, amount of time they awake during the night, and final wake-up time. These parameters were used to calculate total sleep time (TST; time from falling asleep to final wake-up time), sleep onset latency (SOL; time from getting into bed to falling asleep), and wake after sleep onset (WASO; time spent awake during the night). Each morning, adolescents also rated previous night’s sleep quality on an 11-point NRS (0–10) with higher ratings indicating higher sleep quality.

### Electronic monitoring of opioid use

Participants were invited to use an electronic pill cap, eCAP^TM^ (Information Mediary Corporation) to passively monitor opioid use during the first 14 days following surgery. eCAP^TM^ which appears similar to a standard medication bottle cap, fits on a standard medication amber vial and records the time and date when the vial is opened. Participants were mailed an eCAP^TM^-covered medication vial prior to surgery. They were then instructed to place their prescribed opioids in the vial following surgery. No other counseling regarding medication adherence were given to patients and they also did not receive any advice regarding taking their opioid pain medications from the study team. Participants were asked to remove opioids from the vial at the conclusion of 14 days of observations following surgery. eCAP^TM^-covered medication vials were then mailed back to the study team. Our primary outcome of interest was the total number of oxycodone tablets that each participant used during each of the first 14 post-surgical days.

### Covariates

Type of surgery was classified as (1) anterior cruciate ligament reconstruction, (2) medial collateral ligament reconstruction, (3) other knee procedures (e.g. lateral collateral ligament reconstruction, meniscus repair, medial patellofemoral ligament reconstruction), and (4) shoulder and elbow surgery. Oxycodone prescription was categorized as 12 or 18 doses of oxycodone prescribed. Both of these variables, along with biological sex, and time since surgery were included as covariates in the analyses.

### Data analytic plan

First, descriptive data were examined and distribution of variable residuals were examined for violations of normality, and intra-class correlations were examined to determine the appropriateness of using multi-level modeling. Additionally, the four assessments per day of mood and pain were collapsed into a daily mean score per person. To systematically test the within-participants mediating role of pain and mood on sleep-opioid relations, we used multilevel structural equation modeling (MSEM). MSEM mediation data analyses were conducted using Mplus Version 8 [22] using robust maximum likelihood estimation, which accounts for non-normally distributed variables and missing data, and also allows for all regression paths (both within-person and between-person) to be estimated simultaneously (as latent) along with calculating the indirect effect for the mediation. Additionally, variables were portioned into within- and between-person components using person-level centering, where within-person coefficients are interpreted as day-to-day deviations from an individual’s mean score (for sleep, pain, and mood), and between-person coefficients are interpreted as mean scores between people (for sleep, pain, and mood) [23].

**Figure 1.**
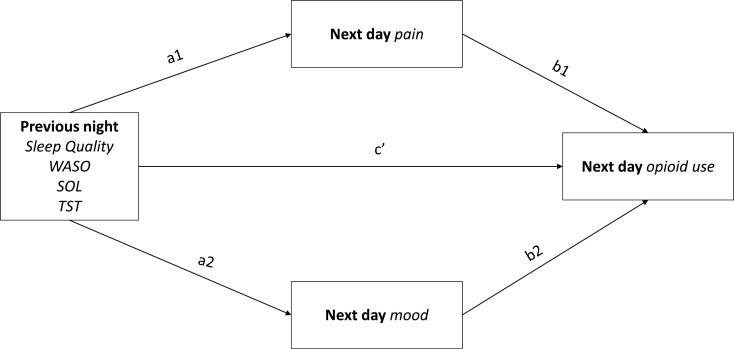
A within- and between-participant model of sleep deficiency, through pain and mood, on opioid use outcomes.

We examined the relationships between previous night’s sleep (self-reported quality, TST, WASO, and SOL) on the number of opioids used per day, mediated by current-day pain and mood, with separate models for each sleep variable. As indicated in Figure 1, we estimated the association between sleep (quality, TST, WASO, and SOL) and pain and mood (mediators; a paths), and the association between pain and mood (mediators) and the number of opioids used (the outcome; b paths). Lastly, we estimated the association between sleep quality (the exposure), and the number of opioids used (the total effect; c path). The indirect mediation effects were calculated as the product of the a and b paths plus the covariance between the slopes (separately for within- and between-person effects). Covariates in the models included day (to account for natural changes in opioid use following surgery recovery), surgery type, biological sex, and the number of opioid doses prescribed post-surgery (12 or 18). Additionally, as an exploratory analysis, all described models were run examining binary daily opioid use (use/no use). Random starts were used for all models to replicate the best log-likelihood. For all regressions, 95% confidence intervals were reported, and statistical significance was set at *p* < .05.

### Sample Size

This study is a secondary data analysis of a larger, ongoing study, including 106 patients enrolled as a non-probability sample; therefore, power analysis was not conducted. The sample size is estimated to be sufficient to test all paths of the mediation models proposed for this study in line with recommendations of Hayes and Rockwood (including at least 10 participants per path to be tested), as well as EMA power simulations, providing support for the study to be sufficiently powered [24].

## Results

### Sample descriptive statistics

The adolescent sample consisted of 106 individuals, 11–19 years of age (mean age = 15 years, SD = 1.8; Table 1). Half of the participants were female (*n* = 55, 52%); most were white, non-Hispanic (*n* = 70, 66%) or American Indian/Alaska Native (*n* = 7, 7%), Native Hawaiian or Other Pacific Islander (*n* = 4, 4%), Hispanic (*n* = 10, 9%), and African-American (*n* = 7, 7%), which is consistent with the demographic profile of families in our region. Most families (75%) had incomes >$75 000 per year. The most common surgical category was anterior cruciate ligament reconstruction (*n* = 32), followed by medial patellofemoral ligament reconstruction (*n* = 28), other knee surgery (*n* = 23), and shoulder/elbow/other surgery (*n* = 23). Across the sample, adolescents rated their sleep quality as moderate at 6.3 (SD = 2.3) out of 10. On average adolescents slept 495 minutes (6 hours and 15 minutes), which is well below recommendations for adolescents (8–10 hours; Table 2). They reported SOL of 30.6 minutes and WASO of 15.7 minutes, which is consistent with other studies among healthy adolescents. Average pain intensity was 2.3 and average mood rating was 6.8 over the 14 days. The average daily number of opioid doses used each day for the 14-day period among the sample was less than one dose per day (0.4 doses per day), ranging from 0 to 5 doses. The average total number of opioid doses used over the 14-day period among the sample was 6.2 doses per participant, ranging from 0 to 34 doses. The intra-class correlations for number of opioids used was 0.85, for pain were 0.30, and for mood was 0.51, providing strong support for the use of multi-level modeling for these data.

**Table 1.** Characteristics of the Sample

Characteristic	Values
*Demographic characteristics*	
Age (years), mean (SD)	15 (1.8)
Sex, *n*(%)	
Male	51 (48)
Female	55 (52)
Race and ethnicity, *n*(%)	
White, non-Hispanic	70 (66)
Black, non-Hispanic	7 (7)
Hispanic	10 (9)
American Indian/Alaska Native	3 (3)
Asian	8 (8)
Native Hawaiian/Pacific Islander	4(4)
Other, non-Hispanic	3 (3)
Missing	1 (1)
Annual household income (US $), *n*(%)	
0–$75 000	25 (24)
$75 001–$200 000	59 (56)
>$20 000	20 (19)
Missing	2 (2)
*Clinical characteristics*	
Surgery type, *n*(%)	
ACL-repair	32 (30)
MCL-repair	28 (26)
Other knee procedure	23 (22)
Shoulder/elbow/other procedure	23 (21)
Opioid doses prescribed prior to surgery	
12 doses	37 (35)
18 doses	56 (53)
Missing	13 (12)

**Table 2.** Descriptive Statistics and Correlation Matrix of Within-Person Study Variables

Variables	Mean (SD)	Sleep Quality	WASO	SOL	TST	Pain	Mood	Opioid use
Sleep quality	6.3 (2.3)	1						
Wake after sleep onset (WASO)	15.7 (40.5)	−0.37***	1					
Sleep onset latency (SOL)	30.6 (38.7)	−0.30***	0.18***	1				
Total sleep time (TST)	495 (102)	0.20***	−0.06*	−0.23***	1			
Pain intensity	2.3 (2.0)	−0.40***	0.23***	0.23***	−0.06*			
Mood rating	6.8 (2.0)	0.52***	−0.17***	−0.15***	0.07*	−0.42***	1	
Opioid use daily	0.4 (1.1)	−0.09***	0.10***	0.06*	−0.01	0.33***	−0.15***	1

*p < .05 **p < .01 ***p < .001.

### Multiple mediation models

#### Model 1: sleep quality.

Multi-level SEM models examined the relationship of sleep quality, through pain, and mood, on opioid use at the within (day level) and between participant levels. Using the within-participant mediation model, we found that sleep quality was significantly associated with pain, and pain was significantly associated with the number of opioids used; the indirect effect of sleep quality, through pain, with the number of opioids used, was statistically significant (b = −0.05, se = 0.01, 95% CI [−0.08, −0.02] *p* = .001). Mood was not a significant mediator (b = 0.0001, se = 0.001, 95% CI [−0.002, 0.002], *p* = .79; Table 3). The within-person total effect pathway, from sleep quality to number of opioids used, was not statistically significant (b = −0.002, se = 0.03, 95% CI [−0.06, 0.06], *p* = .95).

**Table 3.** Within-Person Mediated Effects of Pain Intensity and Mood on Sleep and Opioid Use

Predictors	Mediators	a path (se)	b path (se)	c path (se)	Indirect effect (se)	95% confidence interval	
						Lower	Upper
Sleep quality	Pain intensity	−0.16 (0.04)*	0.30 (0.04)*	−0.002 (0.03)	−0.05 (0.01)*	−0.08	−0.02
WASO		0.006 (0.002)*	0.298 (0.039)*	0.001 (0.001)	0.002 (0.001)*	0.001	0.003
SOL		0.007 (0.001)*	0.299 (0.026)*	0.001 (0.001)	0.002 (0.001)*	0.001	0.003
TST		−0.001 (0.0001)	0.292 (0.026)*	0.0001 (0.0001)	0.0001 (0.0001)	−0.0001	0.0001
Sleep quality	Mood	0.05 (0.04)	−0.01 (0.02)		0.0001 (0.001)	−0.002	0.002
WASO		−0.002 (0.001)	−0.006 (0.20)		0.0001 (0.0001)	−0.001	0.001
SOL		−0.001 (0.001)	−0.009 (0.019)		0.0001 (0.001)	−0.0001	0.0001
TST		0.001 (0.001)	−0.003 (0.019)		0.0001 (0.0001)	−0.0001	0.0001

WASO, wake after sleep onset; SOL, sleep onset latency; TST, total sleep time. c path represents the total effect from sleep variables to opioid use, and is the same for both mediators and only reported once. * indicates statistically significant regression pathway.

For the between-person mediation model paths, sleep quality was significantly associated with pain, and pain was significantly associated with number of opioids used; there was a significant indirect effect (b = −0.07, se = 0.03, 95% CI [−0.13, −0.02], *p* = .01); there was no indirect effect evident for mood (b = 0.0001, se = 0.03, 95% CI [−0.005, 0.008], *p* = .73; Table 3). The between-participants total effect pathway of sleep quality on the number of opioids used, indicates that sleep quality is significantly associated with number of opioids used (b = −0.08, se = 0.04, 95% CI [−0.15, −0.007], *p* = .03).

Exploratory analyses examining the same models with a binary opioid use (yes/no) outcome indicated a significant indirect effect at the within-person (b = −0.100, se = 0.05, 95% CI [−0.192, −0.008], odds ratio (OR) = 0.90, *p* = .03) and between-person (b = −0.382, se = 0.14, 95% CI [−0.651, −0.114], OR = 0.68, *p* = .005) level of sleep quality, through pain, on opioid use odds; no effect was evident at the within-person or between-person level through mood.

#### Model 2: WASO (wake after sleep onset).

For the within-participants mediation model path of WASO, through pain, on number of opioids used, WASO was significantly associated with pain, and pain was significantly associated with the number of opioids used; the indirect effect WASO, through pain, with the number of opioids used, was statistically significant (b = 0.002, se = 0.001, 95% CI [0.001, 0.003], *p* = .002; Table 3); there was no significant indirect effect for mood (b = 0.0001, se = 0.0001, 95% CI [−0.0001, 0.0001], *p* = .79). The total effect pathway indicated that WASO was not significantly associated with the number of opioids used (b = 0.001, se = 0.001, 95% CI [0.000, 0.003], *p* = .14).

For the between-person mediation model paths, WASO was significantly associated with pain, and pain was significantly associated with number of opioids used; there was a significant indirect effect (b = 0.004, se = 0.002, 95% CI [0.001, 0.008], *p* = .02; Table 4). Mood was not a significant mediator (b = 0.0001, se = 0.0001, 95% CI [−0.001, 0.001], *p* = .69). The total effect pathway of WASO on the number of opioids used was statistically significant (b = 0.006, se = 0.002, 95% CI [0.002, 0.010], *p* = .007).

**Table 4.** Between-Person Mediated Effects of Pain Intensity and Mood on Sleep and Opioid Use

Predictors	Mediators	a path (se)	b path (se)	c path (se)	Indirect effect (se)	95% confidence interval	
						Lower	Upper
Sleep quality	Pain intensity	−0.16 (0.04)*	0.30 (0.04)*	−0.002 (0.03)*	−0.05 (0.03)*	−0.08	−0.02
WASO		0.03 (0.008)*	0.128 (0.05)*	0.006 (0.002)*	0.004 (0.002)*	0.001	0.008
SOL		0.03 (0.007)*	0.121 (0.039)*	0.004 (0.003)	0.003 (0.001)*	0.001	0.006
TST		−0.004 (0.003)	0.144 (0.04)*	0.0001 (0.0001)	0.0001 (0.0001)	−0.001	0.001
Sleep quality	Mood	0.10 (0.16)	0.01 (0.03)		0.001 (0.003)	−0.005	0.008
WASO		−0.014 (0.008)	0.011 (0.03)		0.0001 (0.0001)	−0.001	0.001
SOL		−0.011 (0.010)	0.003 (0.03)		0.0001 (0.0001)	−0.001	0.001
TST		0.004 (0.003)	0.007 (0.03)		0.0001 (0.0001)	−0.001	0.001

WASO, wake after sleep onset; SOL, sleep onset latency; TST, total sleep time. c path represents the total effect from sleep variables to opioid use, and is the same for both mediators and only reported once. * indicates statistically significant regression pathway.

Exploratory analyses examining the same models with a binary opioid use (yes/no) outcome indicate a significant indirect effect at the within-person (b = 0.003, se = 0.001, 95% CI [0.0001, 0.003], OR = 1.003, *p* = .02) and between-person (b = 0.018, se = 0.008, 95% CI [0.002, 0.034], OR = 1.02, *p* = .03) level of WASO, through pain, on opioid use odds; no effect was evident at the within-person (b = 0.0001, se = 0.0001, *p* = .70) or between-person level through mood (b = −0.002, se = 0.002, 95% CI [−0.006, 0.003], *p* = .46).

#### Model 3: SOL.

Multi-level SEM models examined the relationship of SOL, through pain and mood, on opioid use at the within (momentary) and between participant levels. For the within-participants mediation model path of SOL, through pain, on number of opioids used, SOL was significantly associated with pain, and pain was significantly associated with the number of opioids used; the indirect effect of SOL, through pain, with the number of opioids used, was statistically significant (b = 0.002, se = 0.0001, 95% CI [0.001, 0.003], *p* < .001; see Table 3). Similar to previous models, mood was not a significant mediator (b = 0.0001, se = 0.0001, 95% CI [−0.0001, 0.0001], *p* = .68). The total effect pathway indicated that SOL was not significantly associated with the number of opioids used (b = 0.001, se = 0.001, 95% CI [−0.001, 0.003], *p* = .28).

For the between-person mediation model paths, SOL was significantly associated with pain, and pain was significantly associated with number of opioids used; there was a significant indirect effect (b = 0.003, se = 0.001, 95% CI [0.001, 0.003], *p* = .017; Table 4); no indirect effect was evident for mood (b = 0.0001, se = 0.0001, 95% CI [−0.001, 0.001], *p* = .92). The direct pathway of sleep quality on the number of opioids used, controlling for all regression paths, was not significant, suggesting the between-person relationship between sleep quality and number of opioids used was fully explained by pain (b = 0.001, se = 0.001, 95% CI [−0.002, 0.009], *p* = .28).

Exploratory analyses examining the same models with a binary opioid use (yes/no) outcome indicate a significant indirect effect at the within-person level of SOL, through pain, on opioid use odds (b = 0.005, se = 0.002, 95% CI [0.001, 0.008], OR = 1.01, *p* = .006); no effect was evident at the between-person level for pain (b = 0.014, se = 0.009, 95% CI [−0.003, 0.031], *p* = .099), or at the within-person (b = 0.0001, se = 0.0001, *p* = .65) or between-person level through mood (b = −0.001, se = 0.002, 95% CI [−0.004, 0.002], *p* = .47).

#### Model 4: TST.

For TST, there was no significant indirect effect at the within or between-person level for number of opioids used, and TST was not independently associated with pain, mood, or number of opioids used. Similarly, for the binary opioid use (yes/no) outcome, there was no significant indirect effect at the within- or between-person level for pain or mood.

## Discussion

In the current study, we evaluated pain intensity and mood symptoms as mediators underlying associations between sleep and opioid use among adolescents undergoing outpatient orthopedic surgery. In support of our hypotheses and past work, we found that greater sleep deficiency was related to higher opioid use at the between-person level. We did not find that greater sleep deficiency was related to higher opioid use at the within-person level. Mediation analyses at both the within-person and between-person level indicated that pain, but not mood, mediated the relationship between sleep deficiency and opioid use. This highlights that when a person reports poorer sleep than their “typical” mean level (as well as poor sleep on average), it contributes to elevated pain, which in turn, leads to higher opioid use. These findings shed light on potential avenues for optimizing pain management and opioid outcomes in this patient population.

Existing prospective, longitudinal studies have found that greater sleep disturbance is associated with increased short-acting opioid use the following day. Raymond et al. used self-reported sleep reports and actigraphy to measure prospective associations between sleep disturbances, pain intensity, and opioid use in 16 hospitalized non-ventilated burn adult patients [13]. Burn patients experienced variable sleep duration with frequent awakenings, averaging around 5.5 hours of sleep per night. More time spend awake during the previous night was linked to higher pain intensity and increased analgesic medication use the following day. While our results are consistent, Raymond et al. did not consider the mediating role of pain intensity and mood symptoms in their analytical models. Mun et al. explored the within-person links between sleep disturbance, pain intensity and pain catastrophizing, and short-acting opioid use in 45 adults sickle cell disease patients taking opioids for pain. Findings revealed that poorer sleep continuity, measured by self-reported WASO, predicted increased short-acting opioid use on the following day [12]. Pain severity and pain catastrophizing mediated this relationship. Our study found direct associations between sleep deficiency and opioid use between participants, but not within the person. It is possible that the impact of sleep deficiency on opioid use is cumulative rather than momentary, such that continuous sleep disturbance over a time period increases general risk within person. Furthermore, significant differences between these previous studies and ours, include that our sample included adolescents with acute (not chronic) pain following surgery. Similarly, to the previous studies, we found strong associations between sleep and pain and pain and opioid use. As noted by Mun et al., we also found small effect sizes of daily sleep quality on opioid usage[20]. Indeed, Mun et al. pointed out that detecting subtle effects through EMA within individuals is expected. There are several hypothesized reasons for this finding including that behaviors and symptoms fluctuate throughout the day, diluting variability, and making it challenging to identify consistent and large effects. In addition, by emphasizing the short-term intra-day correlations, we might potentially downplay the actual long-term impact, since it is feasible that the combined effect over an extended period of time could lead to significant variations in opioid consumption with clinically significant differences in opioid use.

The relationship between sleep deficiency and opioid use is complex and bidirectional, with sleep problems exacerbating opioid use and opioid use impacting sleep patterns [25, 26]. Sleep deficiency may lead to increased opioid use via biological, psychological, and social mechanisms. From a biological aspect, both sleep and opioids have significant effects on the reward system [25]. Sleep plays an important role in maintaining reward system functioning, with disrupted sleep being associated with changes in reward-related brain activity such as altered dopaminergic release (the principal neurotransmitter responsible for reward processing) and receptor sensitivity resulting in reduced sensitivity to pleasurable experiences [25]. Sleep-deprived individuals may seek stronger sources of reward such as opioids. Indeed, opioids activate the brain’s reward system by binding to relevant regions such as the ventral tegmental area and the nucleus accumbens resulting in increased dopamine release [15]. Psychological and behavioral mechanisms include that sleep deficiency is associated with mood disturbances, including increased anxiety and depressive symptoms. Of note, we did not find an association between sleep, mood, and opioid use; however, a large body of literature have found significant associations between sleep and mood symptoms. Opioid receptors are abundant in human emotional processing circuitry and prescribed opioids may provide temporary relief from negative emotions in some, but not all, patients [15]. Social factors via which sleep deficiency may impact opioid use, include that sleep-deprived adolescents may be less likely to seek out or successfully find information on the risks of opioid use, due to cognitive impairment or lack of time. In addition, new-onset perioperative sleep disturbances, might prompt individuals to use opioids as a form of self-medication to improve subjective sleep and manage sleep-related emotional distress symptoms. Indeed, many teenagers who use opioids report that sleep disturbance is one of the factors motivating their continued opioid use [27]. However, opioids disrupt several aspects of sleep, including architecture and timing thereby potentially creating a negative feedback loop leading to long-term problems with opioid use [28].

Our findings are exploratory, however suggest that perhaps we place a greater focus on perioperative sleep deficiency in order to decrease both postsurgical pain intensity and also prescription opioid use [29]. Presurgical child and parent education about the importance of sleep hygiene and the introduction of mindfulness and relaxation techniques are low-cost interventions that could improve sleep quality in many adolescents presenting for surgery [30]. In addition, opioids and general anesthetics are known to disrupt sleep architecture and anesthesiologists could consider the development of intraoperative anesthesia and pain management protocols that take into account postoperative sleep quality, possibly including non-opioid analgesics, dexmedetomidine, and regional anesthesia techniques [29]. Low-impact post-surgical sleep interventions in the hospital and at home could include introducing quiet hours to encourage sleep, and limiting screen time in the evening. For patients at increased risk of postoperative sleep disturbance such as those with a history of chronic sleep disturbance, clinicians could consider incorporating sleep assessments including validated sleep questionnaires or wearable sleep trackers into their pre-operative evaluations as well as their post-operative monitoring protocols. Management of documented perioperative sleep deficiency could utilize existing behavioral and pharmacological strategies. For example, Cognitive Behavioral Therapy for Insomnia (CBT-I) strategies could be adapted for the postsurgical period, while melatonin demonstrates benefits with low side effects in some adolescents with sleep disturbance [31, 32]. Finally, healthcare providers should be mindful of the potential for increased pain and opioid use among sleep-deficient adolescents, and where possible, additional pain management strategies could be considered. These might include non-opioid medications, physical therapy, or other interventions aimed at improving sleep and reducing pain sensitivity. In essence, these findings urge an integrated approach to pain management and sleep optimization, paving the way for potentially reducing opioid prescriptions and mitigating the risk of opioid dependency in this vulnerable age group. Given the intricate interplay between sleep, pain, and opioid use, future research should explore tailored interventions that address these factors simultaneously.

Our study has several limitations that should be acknowledged. First, the study design is observational in nature, limiting our ability to establish cause-and-effect relationships between sleep, pain, and opioid use. Second, the sample size may not be adequately powered to detect subtle differences or effects. Due to the significant expenses and resources needed to conduct this type of study, we were limited to a convenience sample of 106 individuals and our study should be considered exploratory in nature. Regardless, our study sample is much larger than similar studies and findings could assist in the design of larger, better-powered observational studies or a randomized controlled trial focused on changing perioperative sleep. Third, we relied on self-reported measures for sleep quality and timing, introducing the potential for self-report and recall bias. Future studies should incorporate objective measures of sleep deficiency such as actigraphy. Fourth, our study was conducted in a single academic hospital in the Pacific Northwest and findings may not generalize to other settings.

In conclusion, this study adds to the best of our knowledge of the intricate interplay between sleep deficiency, pain intensity, and opioid use by finding that poorer sleep quality is associated with increased pain, which in turn is associated with increased opioid use among adolescents following outpatient orthopedic surgery. Our findings should be considered preliminary, yet underscore the need for a comprehensive and personalized approach to postsurgical pain management, potentially taking into account sleep quality and timing. By addressing sleep deficiency and pain intensity simultaneously, healthcare practitioners could potentially reduce opioid consumption and decrease associated risks. This study provides a valuable foundation for future research, including longitudinal studies with larger cohorts which could provide insights into the temporal dynamics of sleep deficiency, pain intensity, and opioid use.
